# Supplementary material for: Ancient multiplicity in cyclic nucleotide-gated (CNG) cation channel repertoire was reduced in the ancestor of Olfactores before re-expansion by whole genome duplications in vertebrates
Source: PLoS One. 2022 Dec 30;17(12):e0279548. doi: 10.1371/journal.pone.0279548 (PMC9803222; doi:10.1371/journal.pone.0279548)

Ancient multiplicity in cyclic nucleotide-gated (CNG) cation channel repertoire was reduced in the ancestor of Olfactores before re-expansion by whole genome duplications in vertebrates

Supplementary figures

Figs S1-S17: Phylogenetic trees of the neighboring gene families of *CNGA* and *CNGB*. The trees were made using the same parameters in IQ-Tree as described in Materials and Methods. Clades are colored after the chromosomal location of the closest spotted gar ortholog. Gene names shown are the names of the human proteins in that clade. Solid colors indicate that the clade is considered supported in both aLRT and UFbootstrap analyses. Nodes are considered strong when they have an aLRT supports ≥ 80 % and an ultra-fast bootstrap support ≥ 95 %. Well supported nodes are labelled with a filled red circle.

Fig S18: Phylogenetic trees of actinopterygian fish CNGA (A) and CNGB (B) sequences identified in the NCBI RefSeq protein database through reciprocal BLASTP searches. The trees were made using the same parameters in IQ-Tree as described in Materials and Methods. Clades are colored after the chromosomal location of the closest spotted gar ortholog and zebrafish ortholog. Gene names shown are the names of the human proteins. Nodes are considered strong when they have an aLRT supports ≥ 80 % and an ultra-fast bootstrap support ≥ 95 %. Well supported nodes are labelled with a filled red circle.

Fig S19: The phylogenetic tree presented in Figure 1A, but with a heatmap showing the presence (blue) or absence (red) of domains of vertebrate CNGA sequences. Vertebrate sequences have been labelled with red branches.

CNGA neighbours

Figures S1-S7

S1: ENSFM00250000000562 - ELF

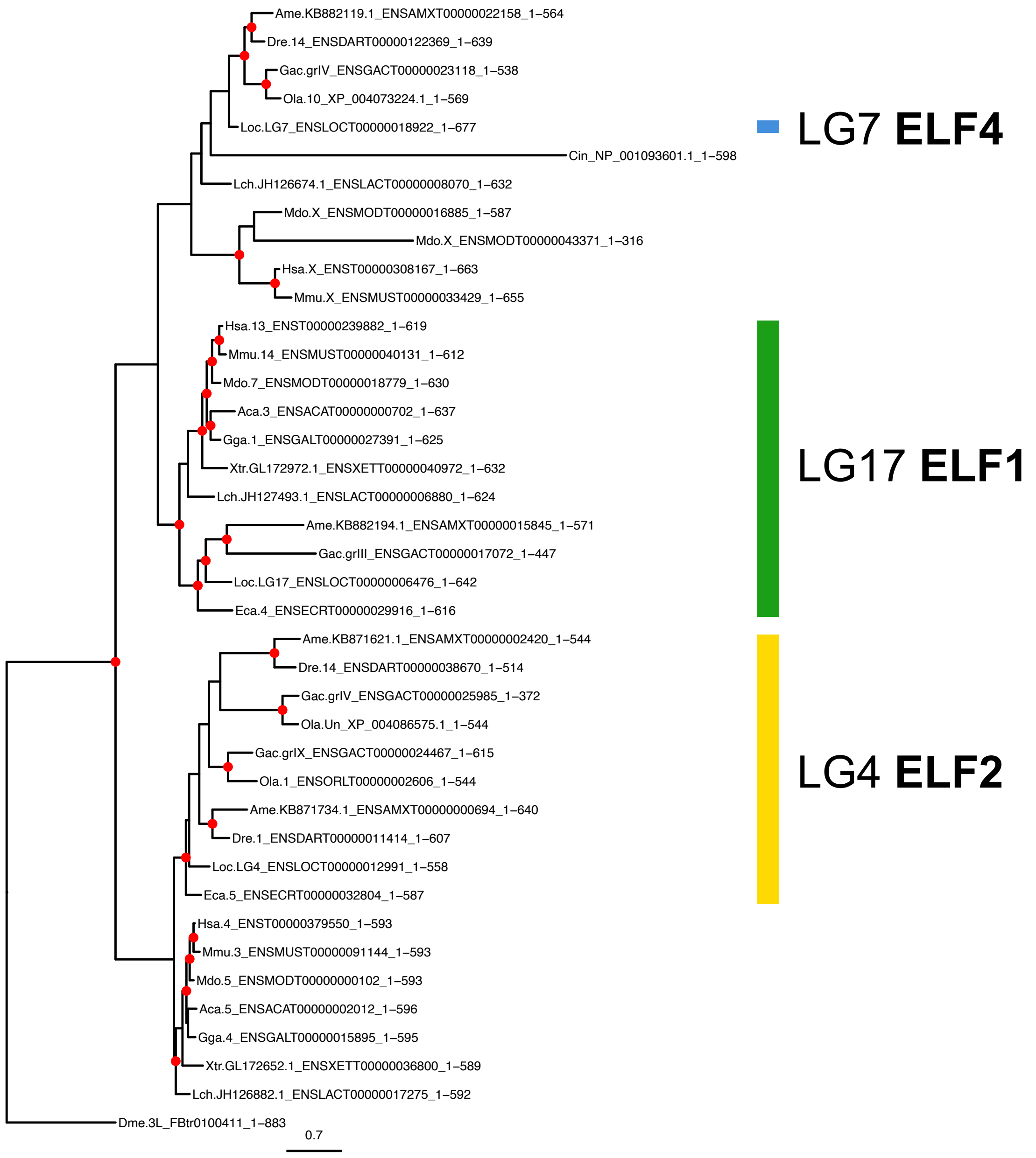

S2: ENSFM00260000050545 - KCTD

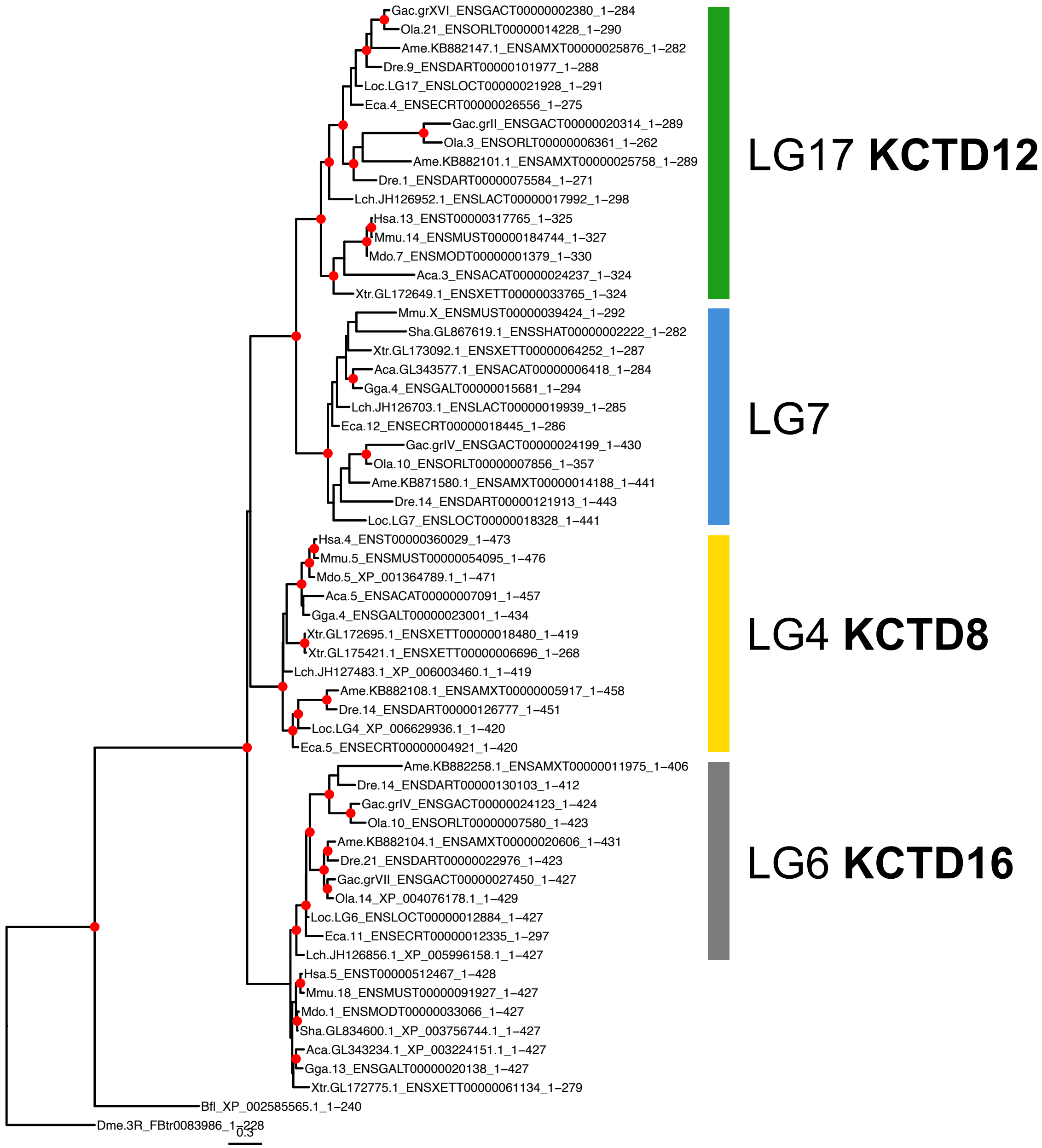

S3: ENSFM00280000058686 - BMX

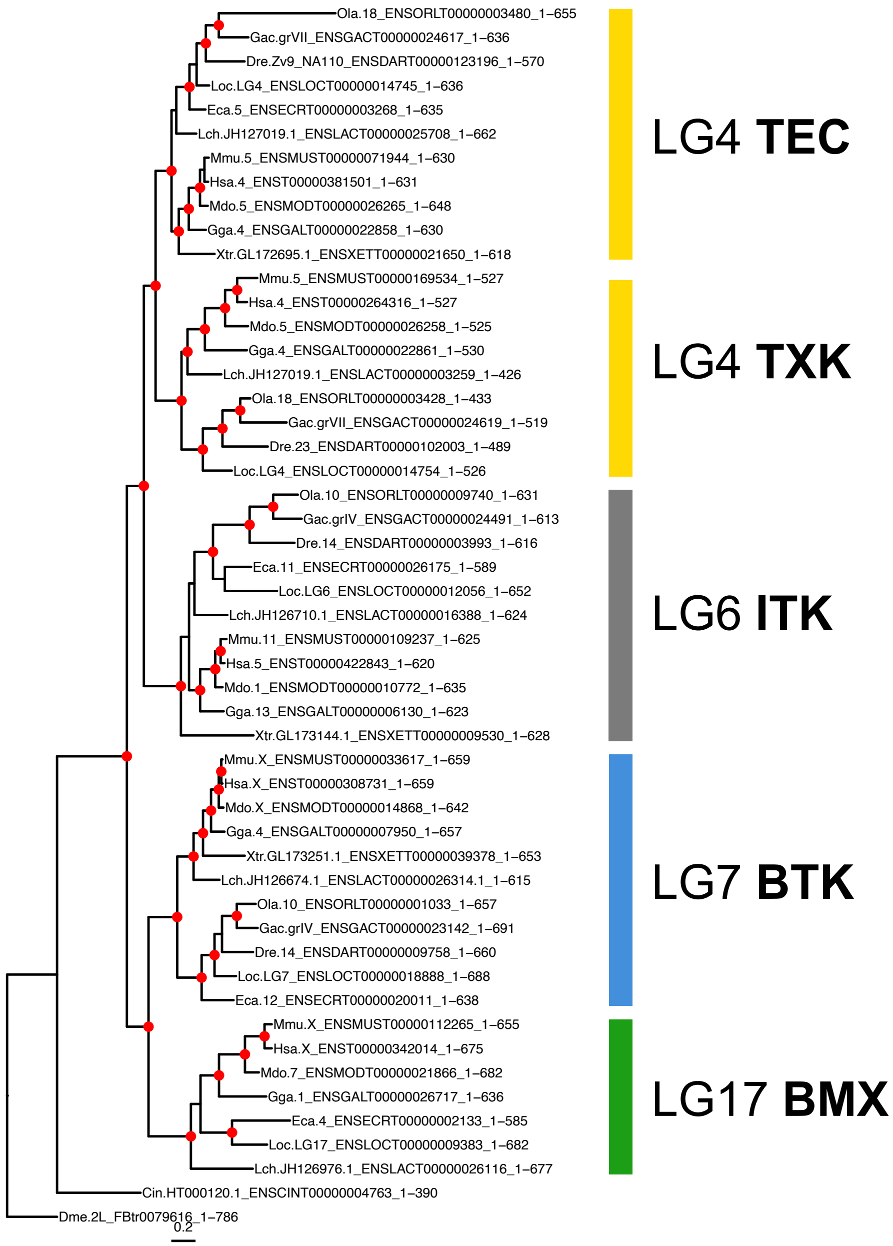

S4: ENSFM00500000269879 - EDNR

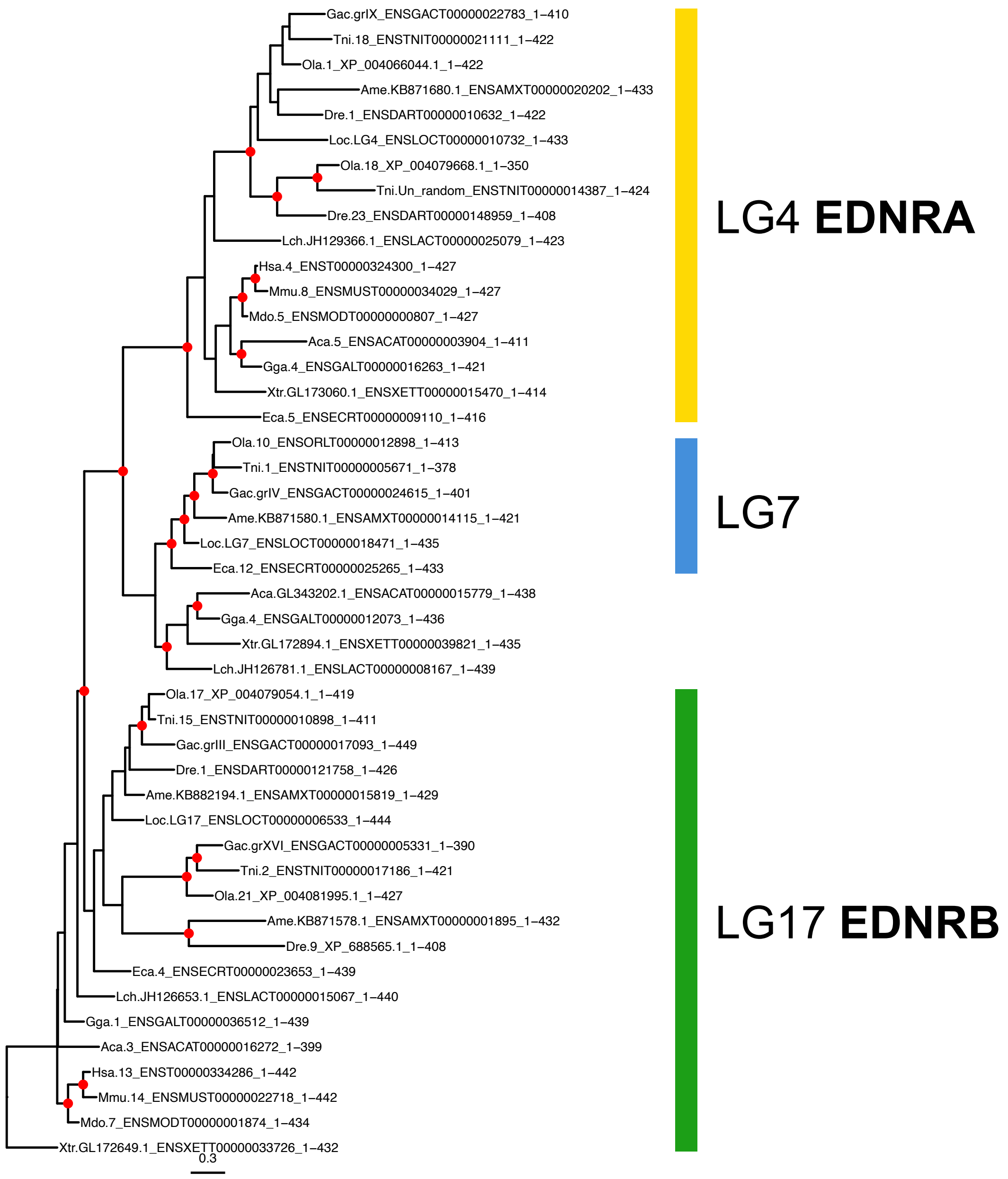

S5: ENSFM00500000270315 – RAB33

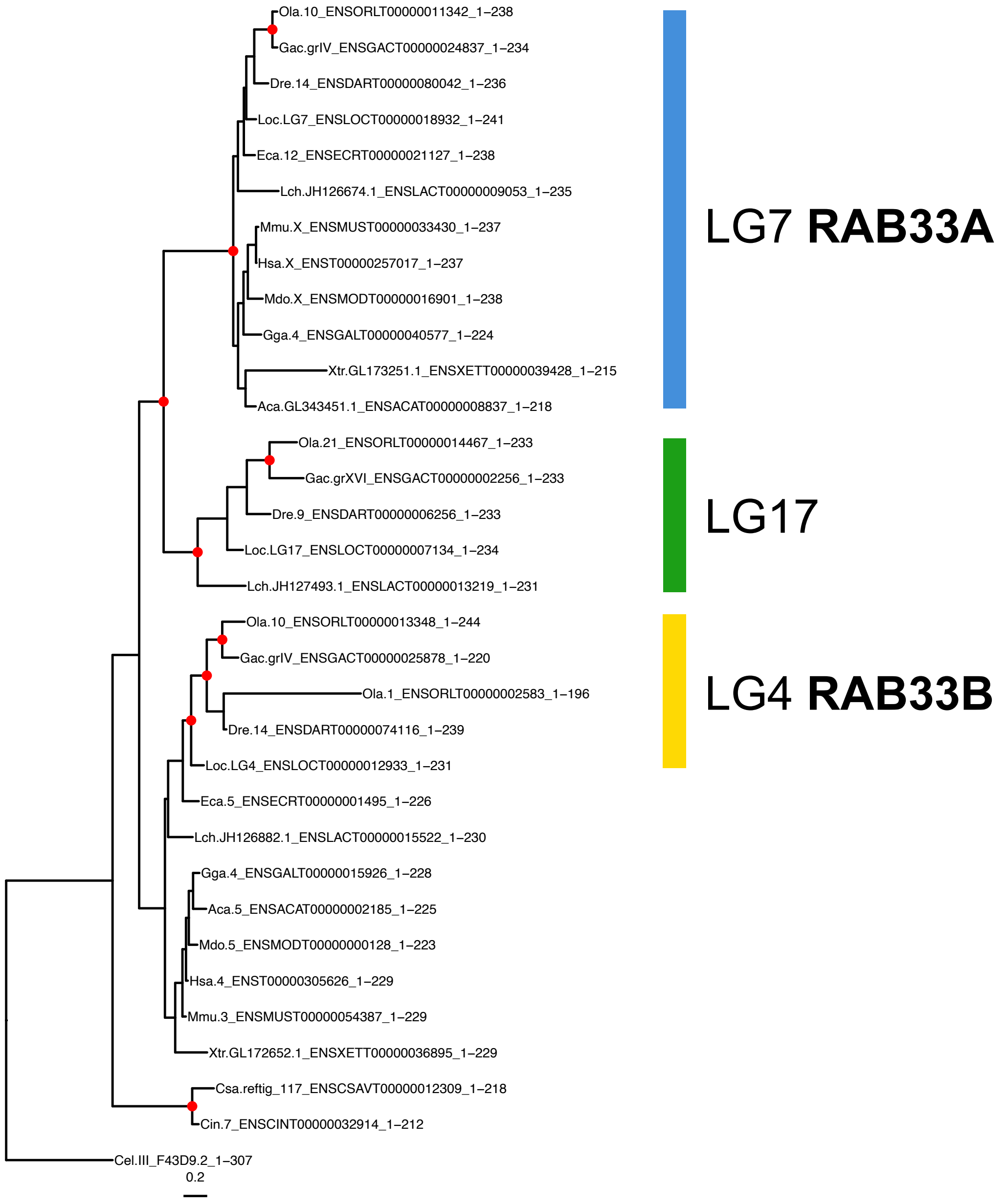

S6: ENSFM00730001521426 - PCDH

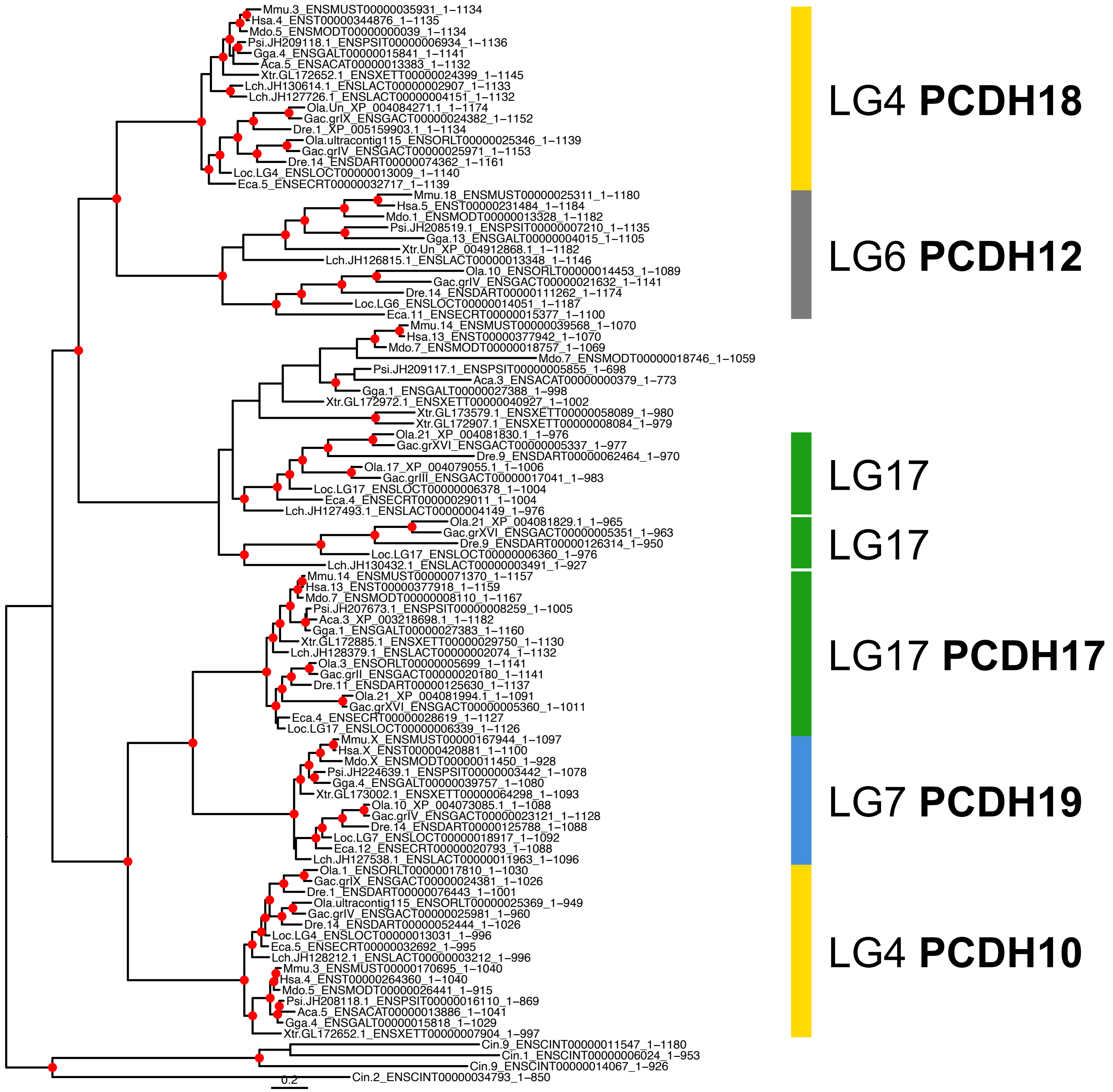

S7: ENSFM00730001521603 - NIPA

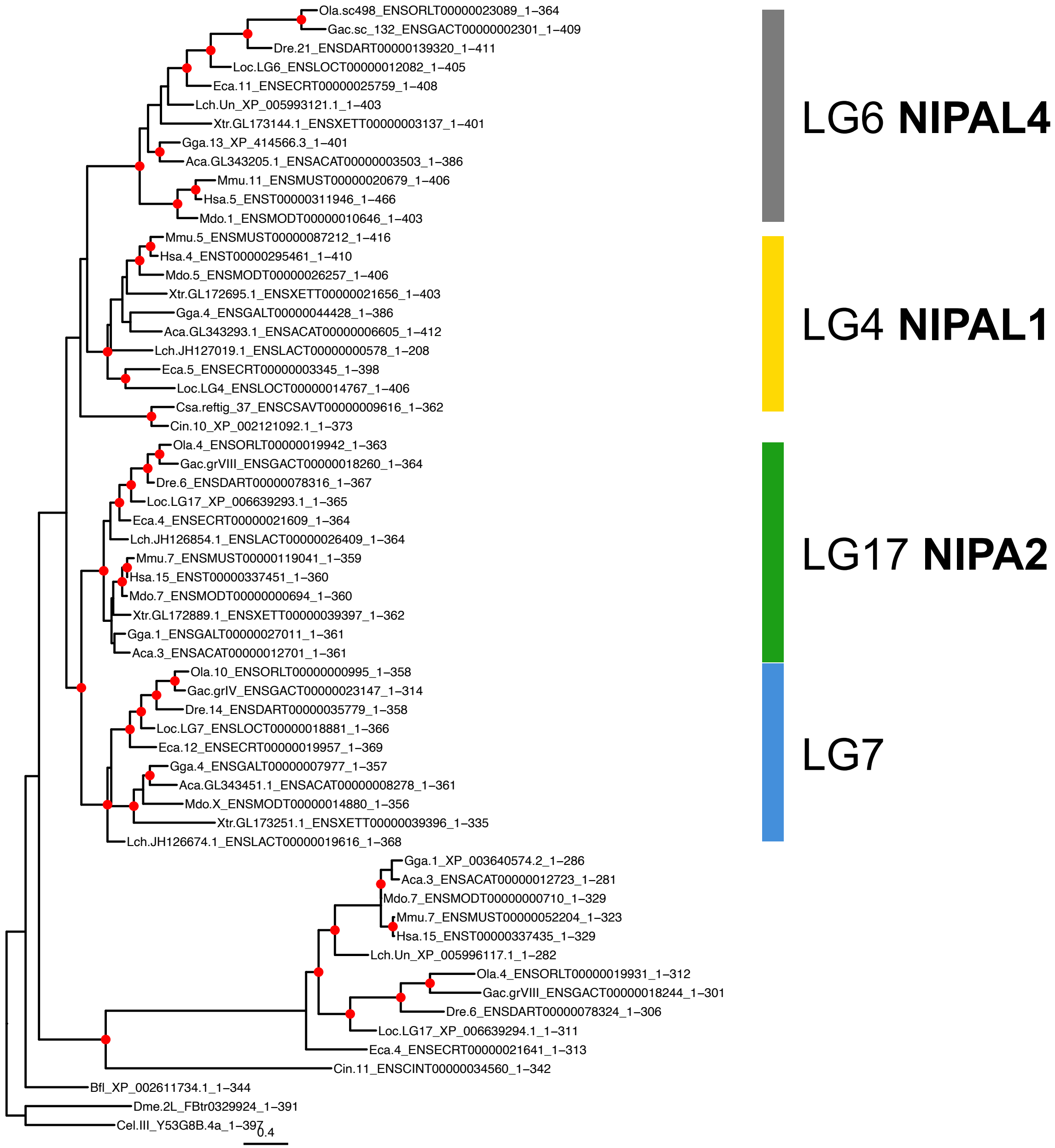

CNGB neighbours

Figures S8-S17

S8: ENSFM00250000000331 – SLC7A

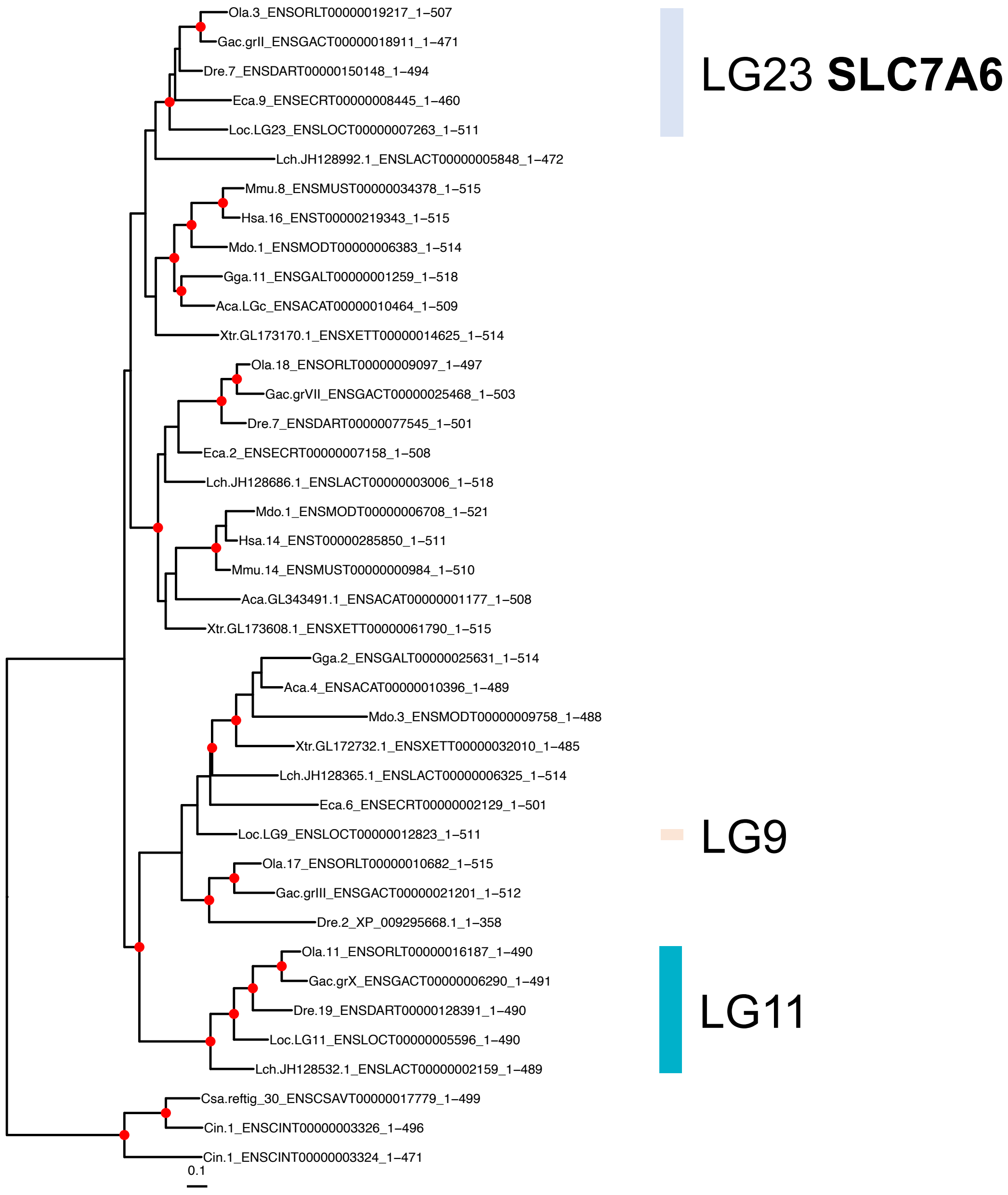

S9: ENSFM00250000001569 - RANBP

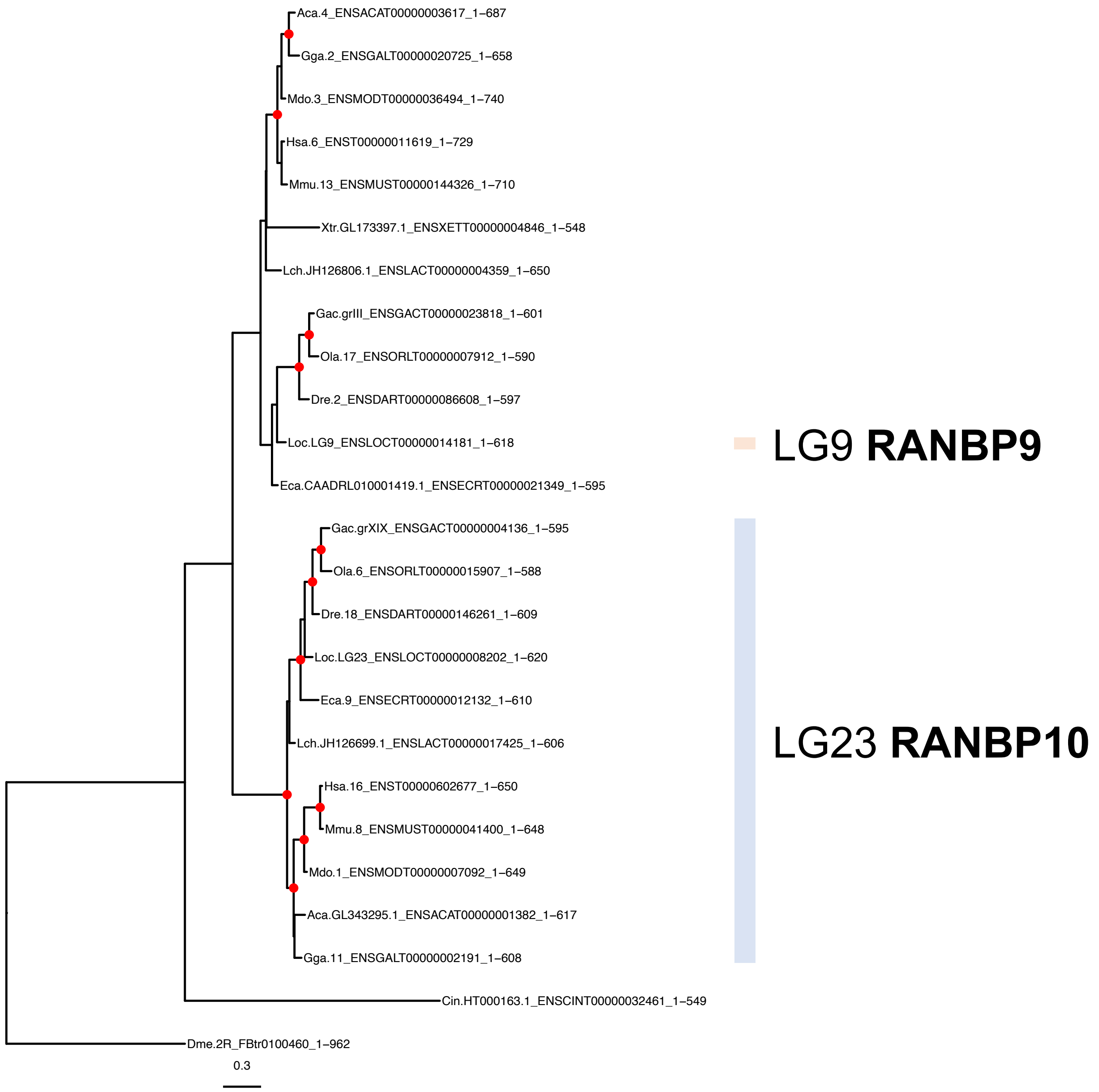

S10: ENSFM00250000001904 - PHLPP

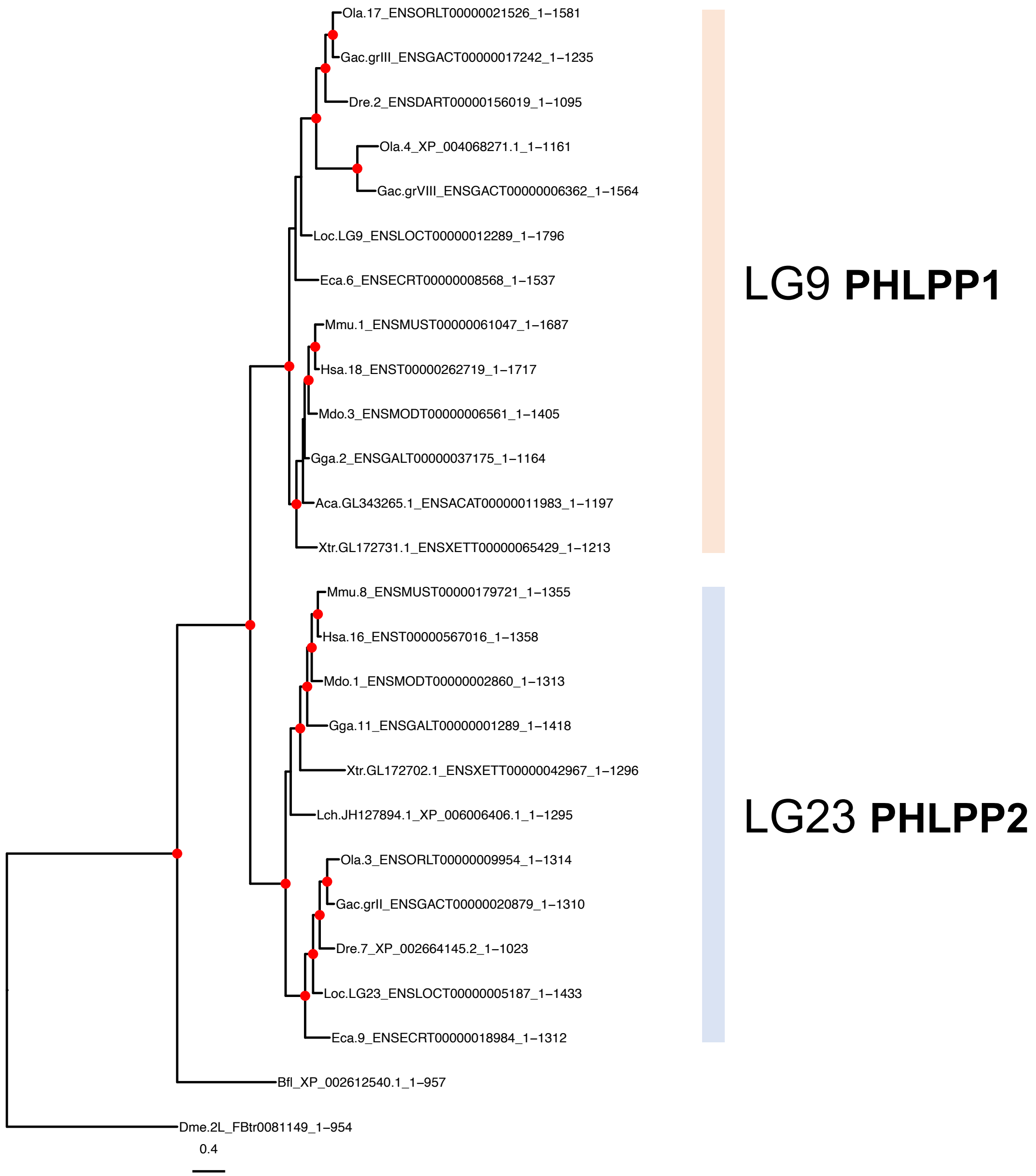

S11: ENSFM00250000002105 - GPT

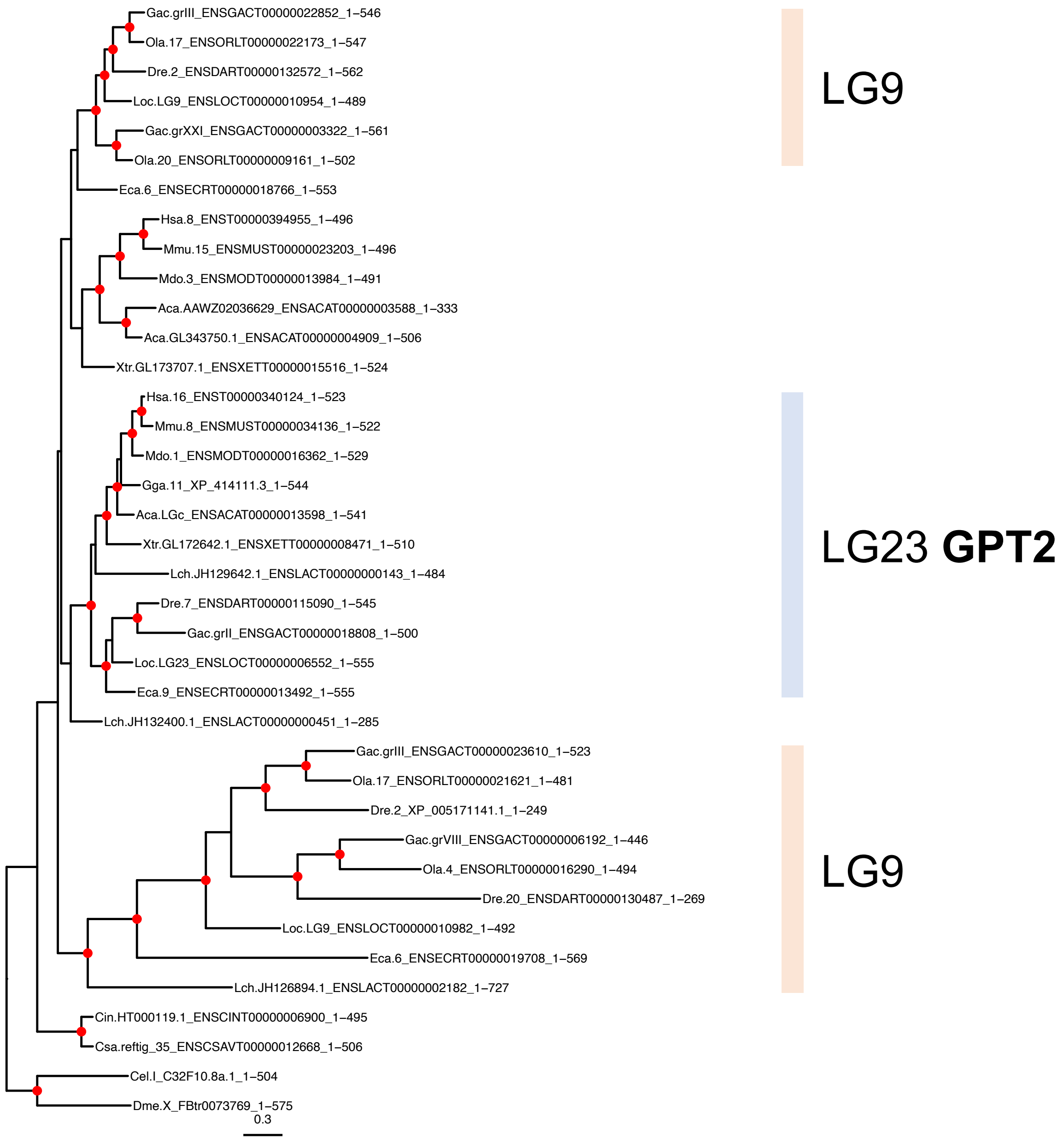

S12: ENSFM00250000003242 - ATP6V0D

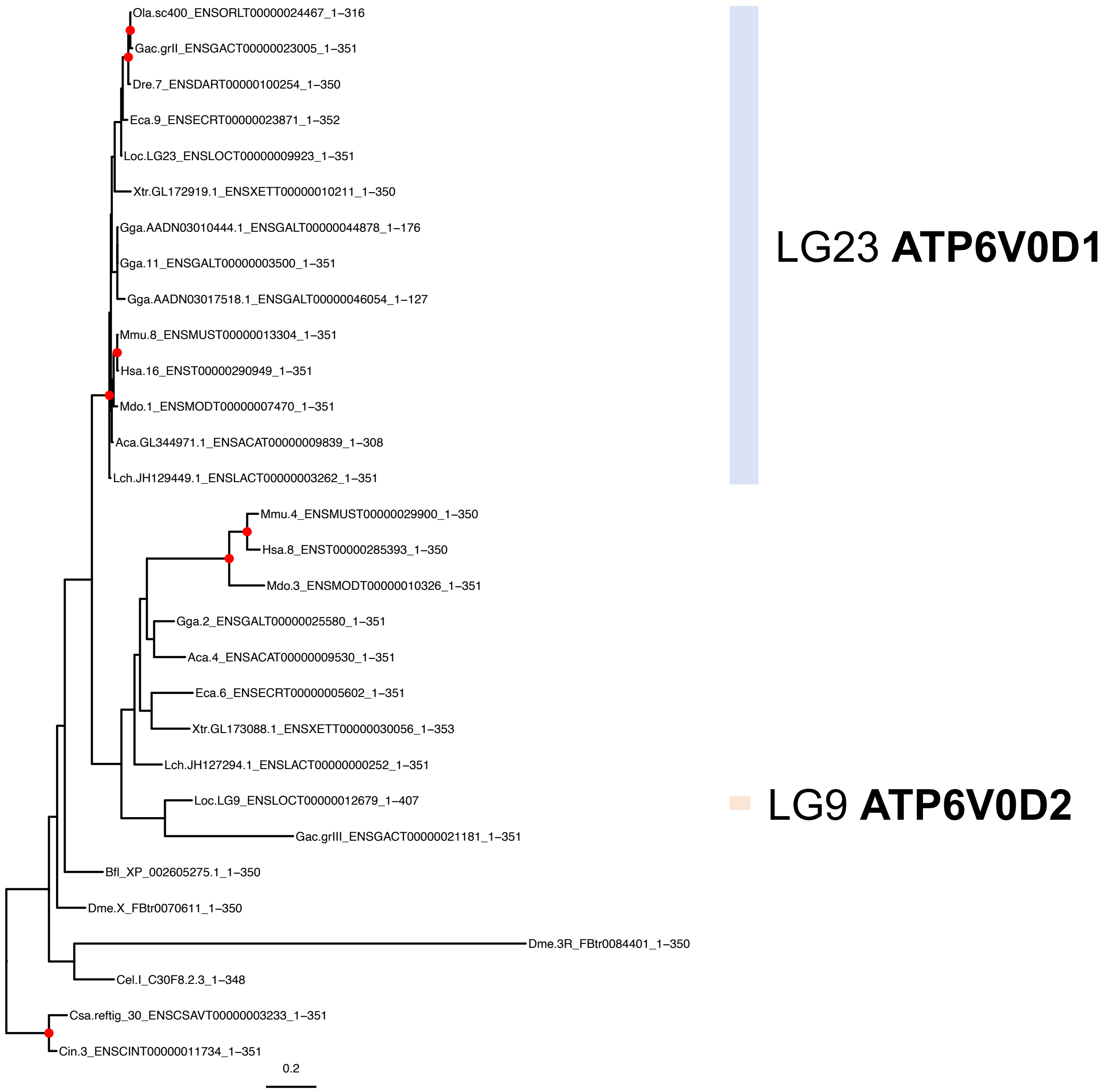

S13: ENSFM00250000003657 - GFOD

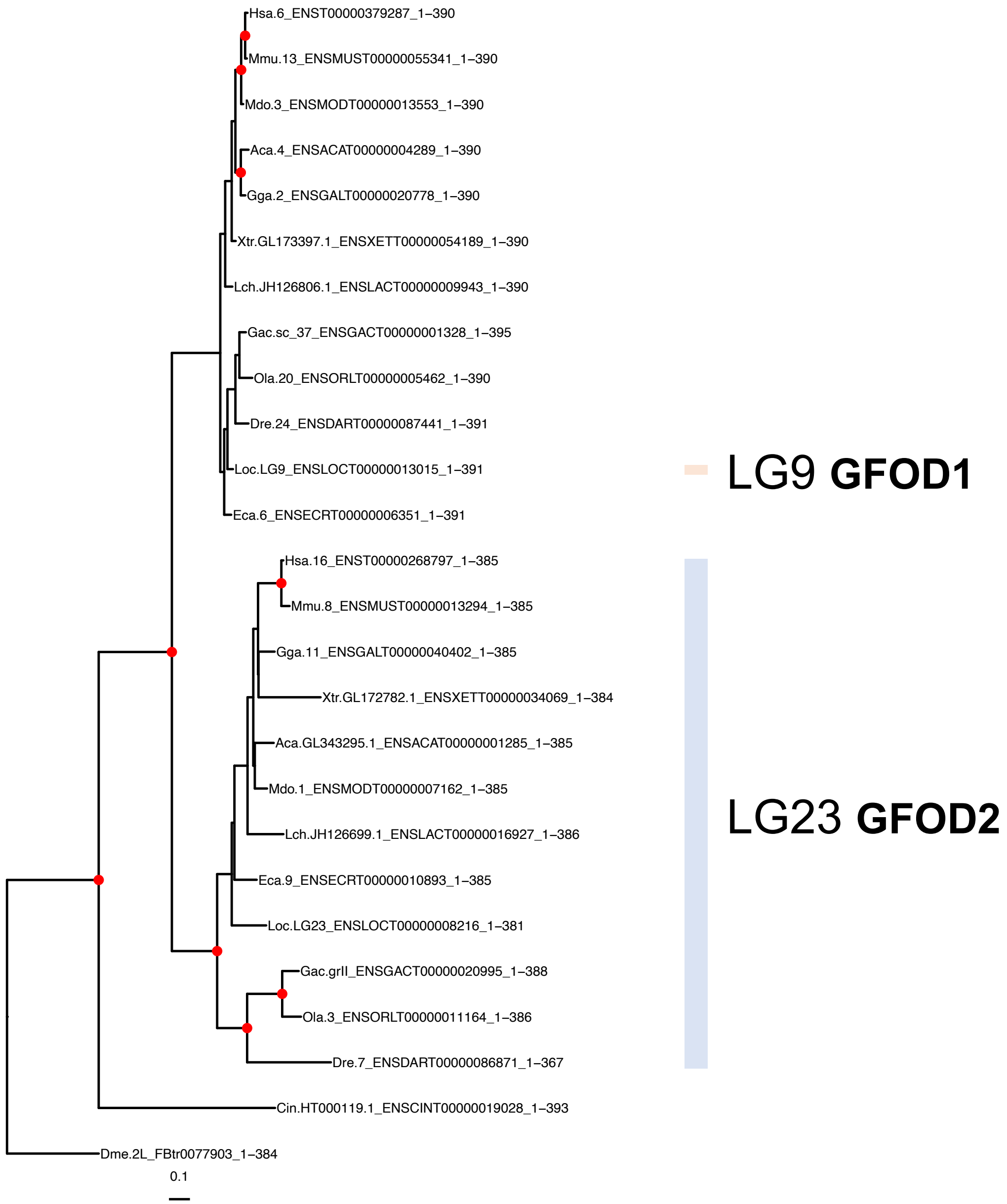

S14: ENSFM00260000050376 – SLC12A

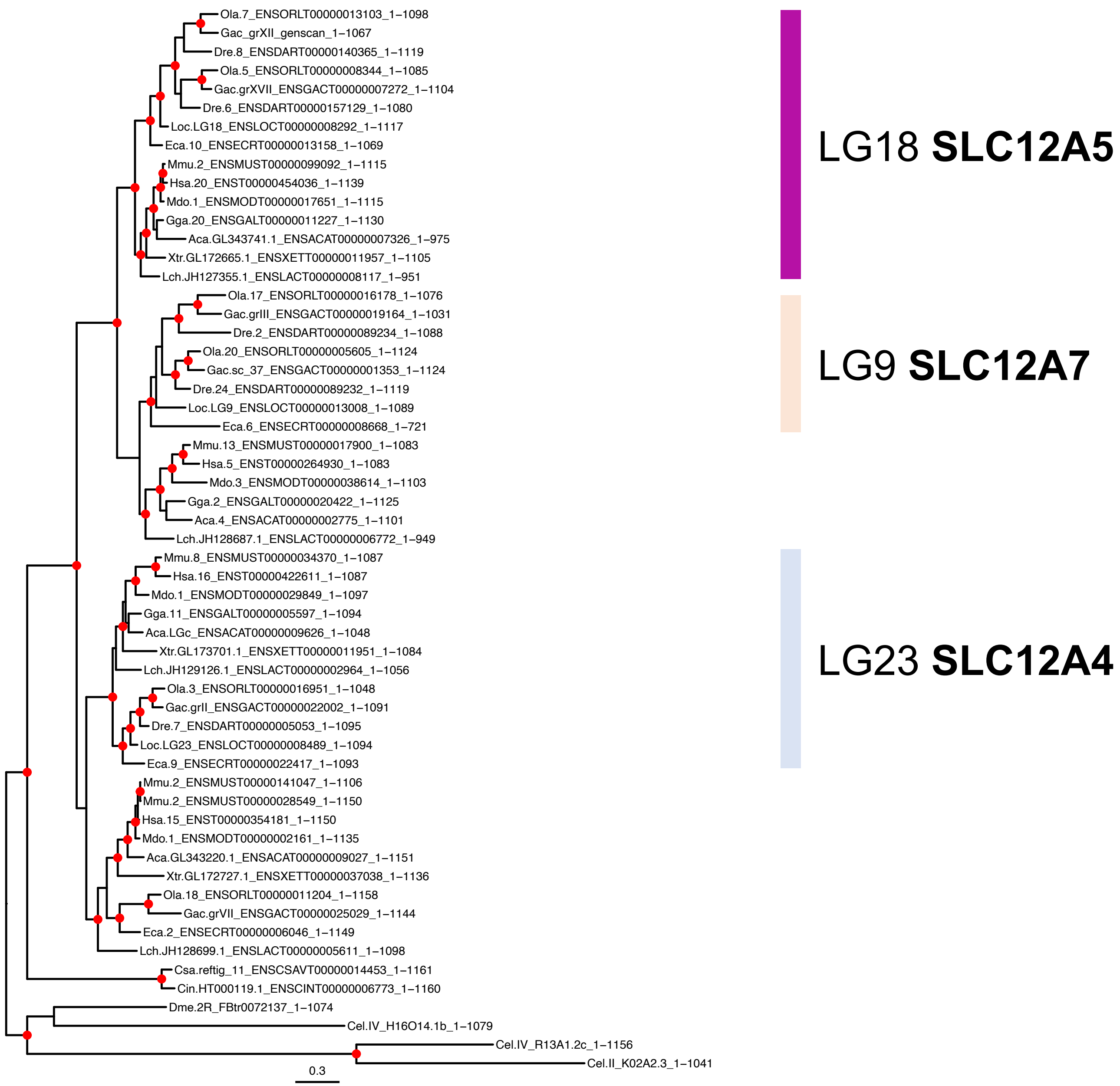

S15: ENSFM00400000131720 - MMP

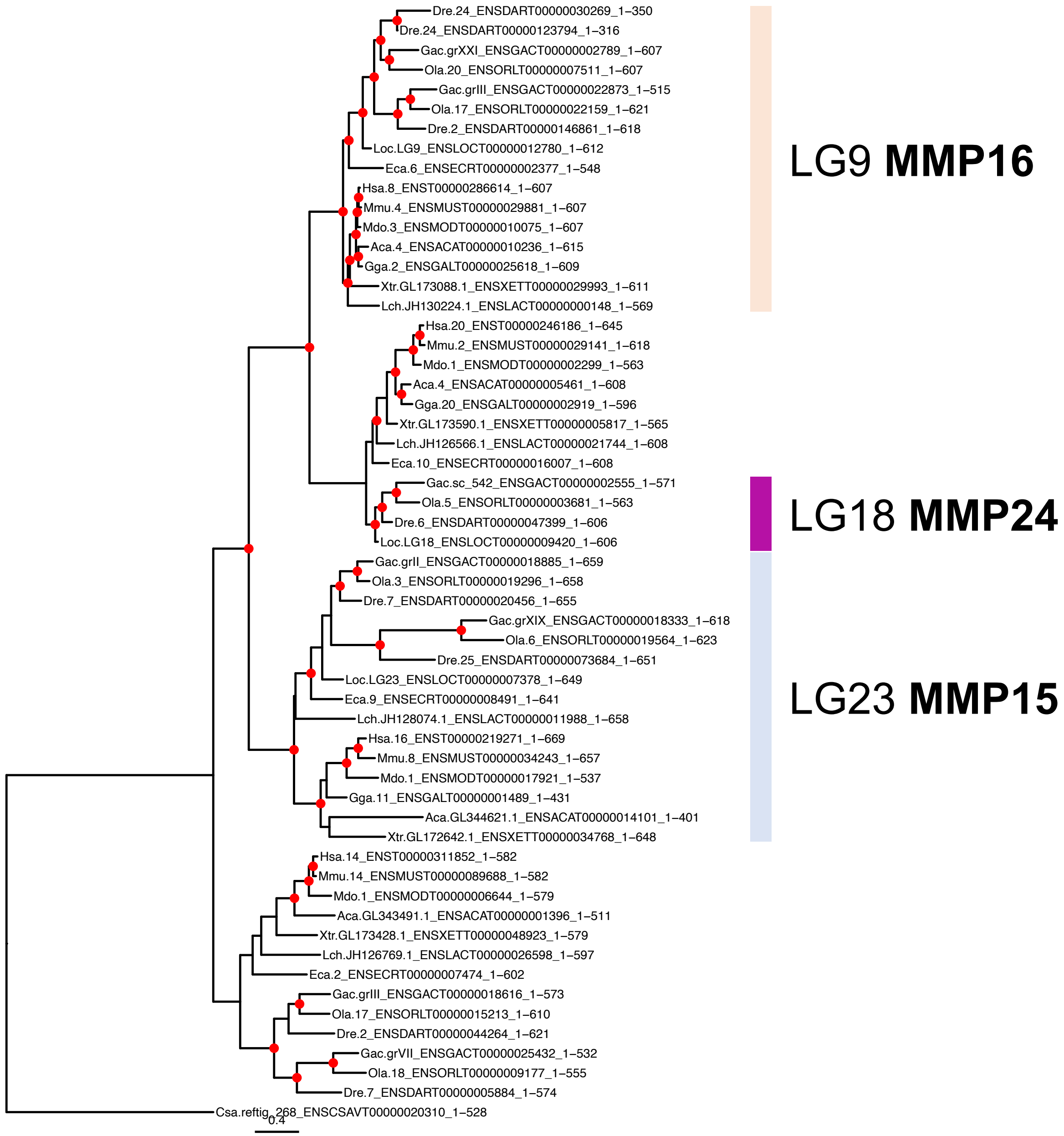

S16: ENSFM00730001521337 - CPNE

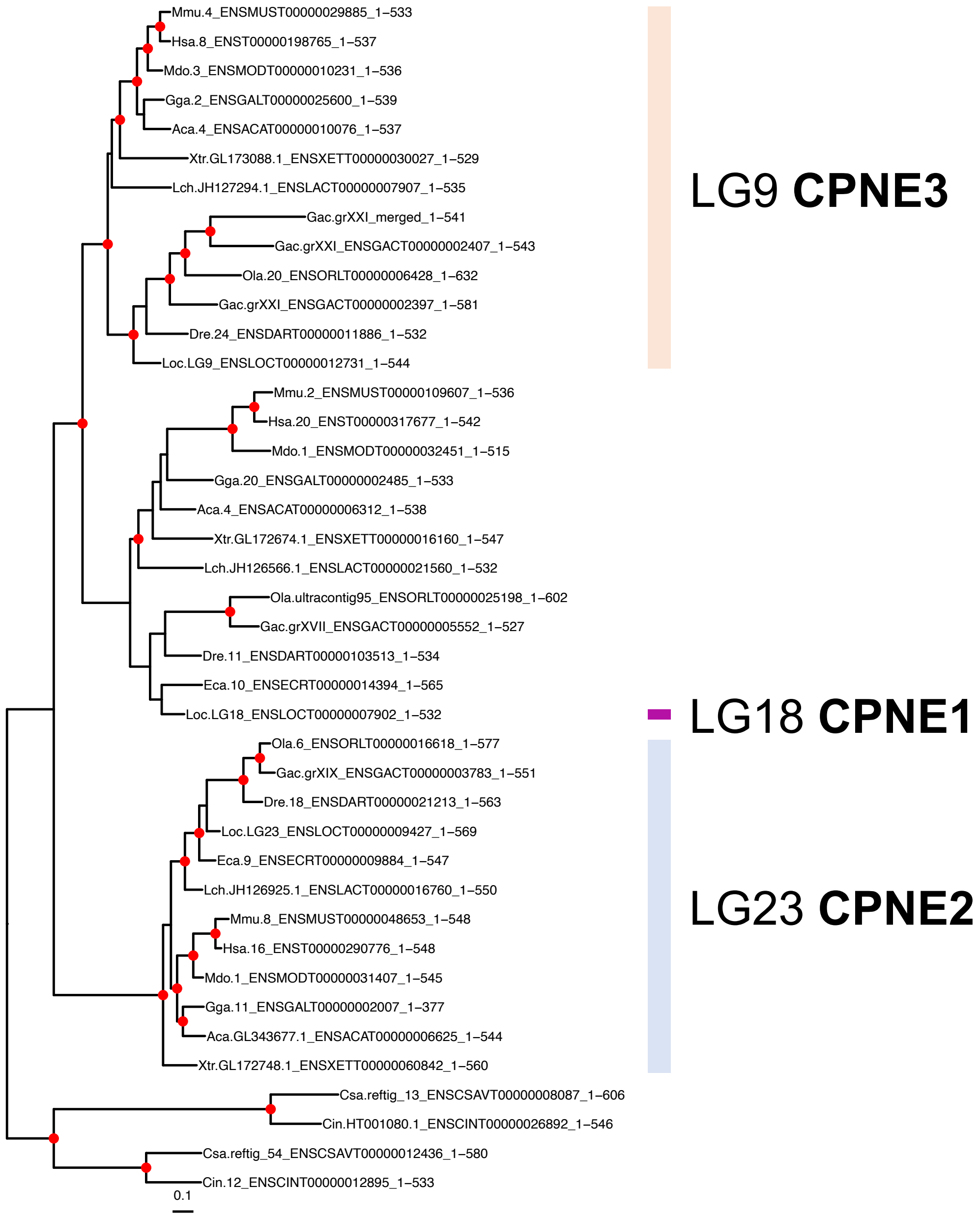

S17: ENSFM00730001521655 - WWP

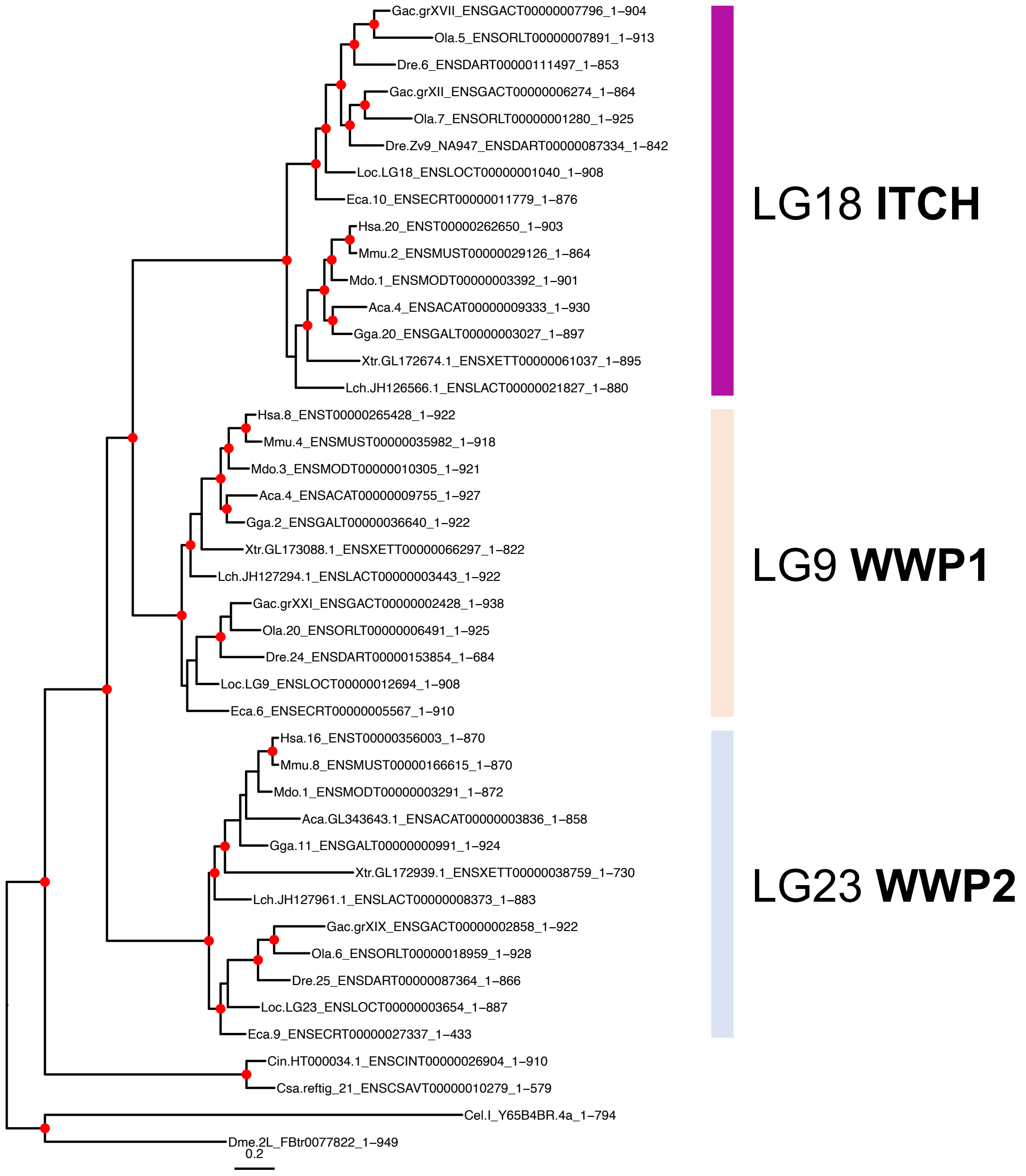

S18:

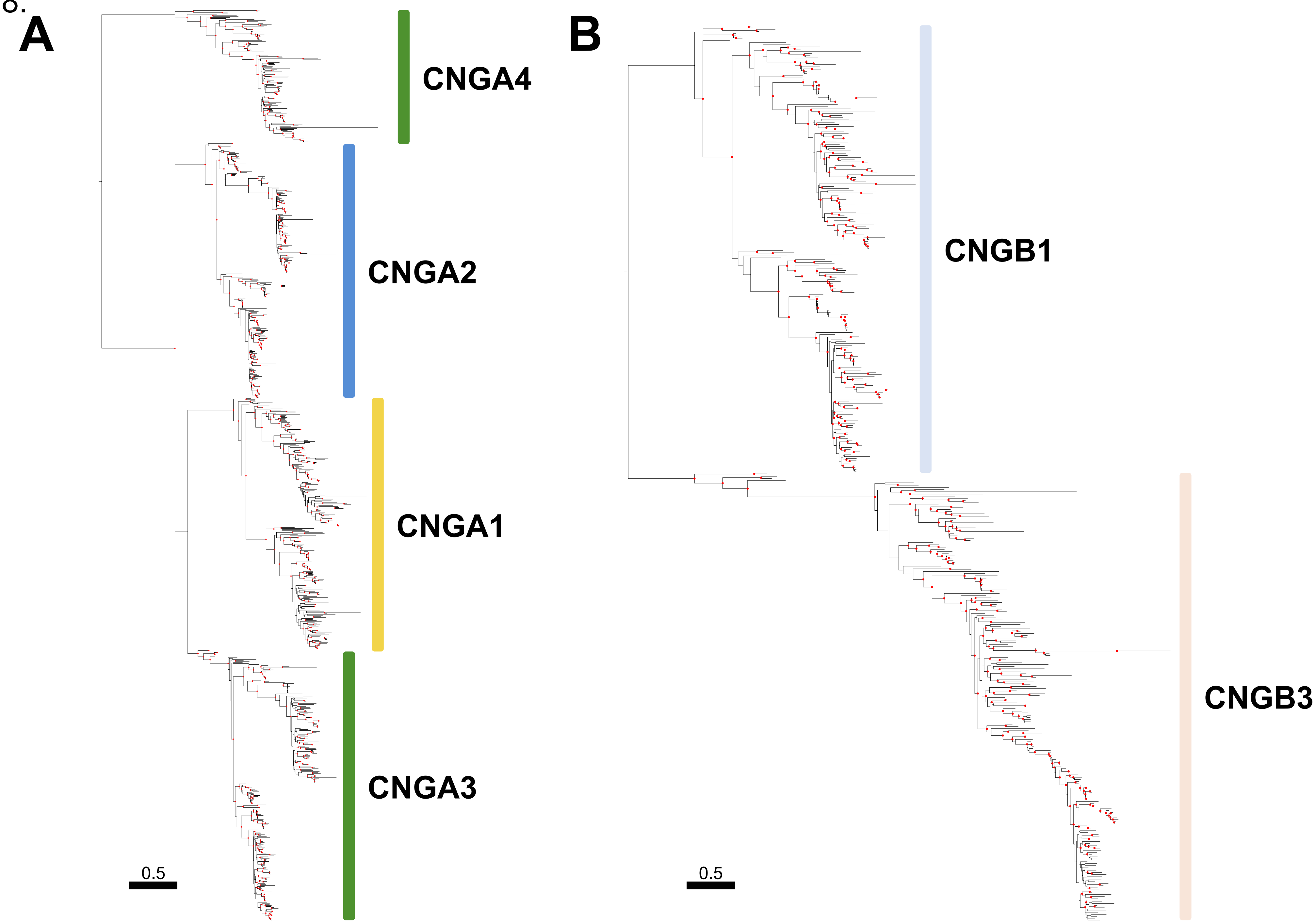

S19:

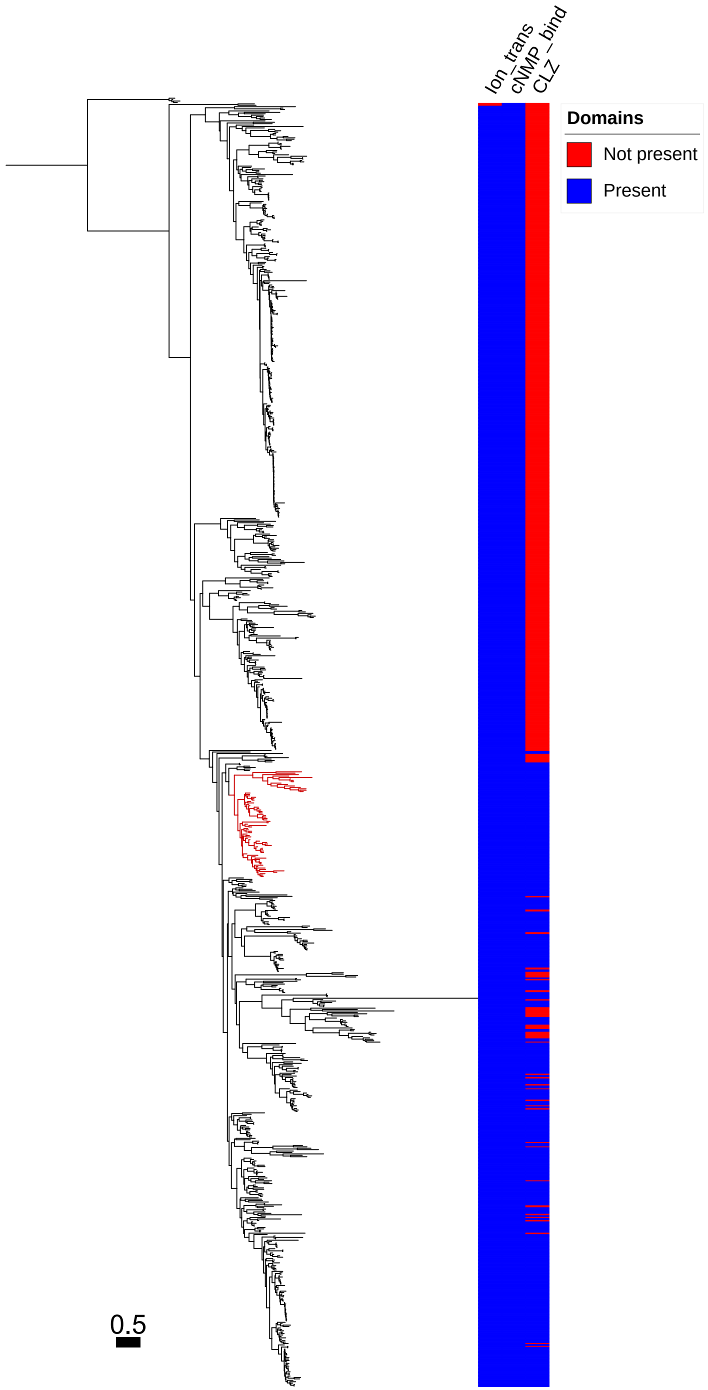

Supplement: S1 File — (PDF) [file pone.0279548.s001.pdf]
