# Supplementary material for: Ancient multiplicity in cyclic nucleotide-gated (CNG) cation channel repertoire was reduced in the ancestor of Olfactores before re-expansion by whole genome duplications in vertebrates
Source: PLoS One. 2022 Dec 30;17(12):e0279548. doi: 10.1371/journal.pone.0279548 (PMC9803222; doi:10.1371/journal.pone.0279548)
Supplement: Attachment — Submitted filename: Response to Reviewers.docx [file pone.0279548.s004.docx]

*We wish to thank the reviewers for sharp-eyed reading of our manuscript and for many constructive suggestions.*

Reviewer #1: Lagman et al present a very detailed and thorough study of the evolution of Cyclic nucleotide-gated cation channels. The authors do a very nice job of putting the results in context both of Metazoan genome evolution, and of the evolution of vision. Overall this is a good focused study of molecular evolution, and I only have a few comments.

Detailed comments:

The authors use the term "invertebrate" many times. This is misleading because as invertebrates are paraphyletic, there is no generalization which covers their evolution and does not also concern vertebrates. When the aim is to study more general metazoan evolution, relaxing the focus on vertebrates, the authors should specify this, and when specific groups are concerned (e.g. metazoans which do not have eyes), they should also specify it. In each case of use of the tern invertebrates, a better term can be found by asking which evolutionary question is being answered, and formulating it clearly.

*Normally, we aim to keep the use of paraphyletic terms to a minimum, but in this case, we failed and thus thank the reviewer in spotting this. We agree with the reviewer and have thus changed the term to metazoans since it works well in the text.*

In the Introduction, the opening statements need references, especially the first sentence. I don't think that Darwin mentionning the eye in 1858 would be sufficient. Is there evidence that vision was one of the most intensively studied topics?

*We agree that this statement was vague and hard to trace, thus we have changed the formulation to that it instead is a topic that has fascinated evolutionary biologists since the days of Darwin, with a reference to the origin of species.*

p. 5, the last paragraph on insight into medical conditions is neither convincing nor needed to make the point of the manuscript, I suggest removing it.

*After consideration we agree with the reviewer and have removed this statement. We chose to keep the paragraph about the mutations, since we think that this is part of an important history in the original identification of these genes and their role in vision.*

p. 11 and following: it would be helpful to present in a figure or table which of the species or lineages sampled use vision "as an important sense" (e.g. most vertebrates, most insects, cephalopods), which use it as a secondary sense, and which do not have vision.

*Since we rephrased according to the suggestion of reviewer number two, and just say “a lineage with a diverse and rich opsin repertoire”, we think such a figure or table is not necessary.*

It would be interesting to compare the vertebrate and especially fish synteny results with the results computed in the specialised tool Genomicus: [https://www.genomicus.bio.ens.psl.eu/genomicus/](https://www.genomicus.bio.ens.psl.eu/genomicus/)

*The Genomicus tool is useful for comparing the genomic neighborhood of a particular gene between several species. However, for our analysis we want to investigate the paralogous regions harboring the CNGA genes. This analysis is possible in Genomicus*, *but it would be cumbersome since we would have to perform much of the analysis manually. Additionally, the data in our analysis is based on is the same original data as Genomicus, thus the results would be similar in the end. In our analyses we inspect the neighboring family alignments, curate when necessary and blast for missing genes, something the Genomicus tool lacks since it is based on the alignments and trees displayed in the Ensembl browser. These alignments and trees are known to sometimes miss some genes in some species or include incomplete sequences that could make the phylogeny of a gene family somewhat different.*

*Finally, the Genomicus karyotype tool does not, as far as we understand, include the genes of our interest all the way back to the chordate ancestor.*

In the methods, please specify the flavor of Blast (sometimes specified as BlastP, sometimes just "Blast"), the version and the parameters used.

*This was unintentionally left out and this information has been added as well as version numbers for CD-HIT.*

Why use reciprocal Blast rather than an ortholog cluster algorithm on all potential homologous genes from the first round of Blast hits?

*Reciprocal blast searches were performed due to the lack of experience using these tools at the time of analysis. If we would redo the analysis, we agree this would be a good option.*

Figure 5: it is not possible to distinguish the closely related colors, which makes the figure very difficult to read and interpret.

*We have now changed the color scale of figure 5, and hope that it makes the figure easier to interpret.*

Legends of Fig 6-8: please specify the color code the first time it is used, i.e. Fig 6.

*This information was provided in the figure legends but has been clarified.*

Typos:
line 74 "an origin"

*This has been fixed.*

line 539 "extend" not "extending"

*This has been fixed.*

line 451 should be same sentence grammatically.

*This has been fixed.*

The following data is "not publicly available" on Figshare: CNGB_actinopterygian_tree.

*This was a mistake in the data upload process and has now been corrected.*

Reviewer #2: The manuscript presents a detailed and very interesting study of the evolution of CNGA and CNGB genes, and clarify duplication and loss processes that led to the current gene repertoires in metazoan species. This study is very complete, combining the analysis of a huge amount of publicly available genomic and transcriptomic data. The literature used as reference is rich and its combination with a well written introduction helps a lot in understanding the results and the discussion. However, in my opinion, the manuscript would gain in clarity after some modifications of the main text and figures and a restructuring.


Suggestions concerning figures:

- I would suggest the authors to color gene phylogenies branches according to the species clade they belong to. This would allow a better understanding of the results, especially for Figure 2, Figure 6 and Figure 7. This is not needed for figure 7 as species clades are already indicated.

*We initially considered doing this but decided for the solution to use figure 2 and figure 3 in combination. The reason for this decision was the issue about where to draw a line between clades, at species, genus, or family, etc., due to the vast number of species and sequences included. For figure 4 the clades were colored based on the deepest supported node.*

- Merge Figure 1 and Figure 3 to a unique figure. One idea: Keep the figure 3 as it is, but adding a phylogenetic tree connecting species clade at the left of the figure (in the same way as what was done for figure 1), and add a column for the presence/absence of CNGB gene. To keep the information about the presence/absence of the cNMP binding domain, and the CLZ domain, a symbol in the boxes can be added.

*We agree with the reviewer that this is a good idea and have merged these figures into figure 3.*

- Figure 5: Add species names in addition to the silhouettes.

*These have been added.*

- Figure 5-E: Removing the circles when no expression is detected, instead of small circles, would improve the figure readability.

*This has been done.*

- Figure 8: Indicate what the number written in the gene boxes represent, either in the figure or in the legend.

*This information has been added to the figure legends.*

- Figure 9: Indicate with an arrow the teleost specific whole genome duplication, in the branch leading to the zebrafish.

*We thank the reviewer for spotting this mistake, this arrow has been added to the figure.*

- Move Table 1 to supplementary table.

*This has been done.*

Suggestions concerning the main text:

- Line 46: “Led to us to” -> “Led us to”

*This has been fixed.*

- Line 137 to line 146: Replace “We identified five clades” to “We identified five CNGA type gene clades”
Here is a proposition for the rest of the paragraph, that would gain in clarity by being rewritten:

“We propose a naming for these five clades : - CNGA (named upon traditional CNGA sequences) in which most genes have a CLZ domain and containing representative sequences of vertebrates and sponge ; - CNGE which is only found in cnidarian ; - CNGF which is present in bilaterian and cnidarian but which have been lost in deuterostomes ; -CNGC present in both Ctenophore, Cnidaria and some Bilateria clades ; -CNGD which is found in most of metazoan excepted in sponges and placozoans “

*We agree that this makes the paragraph much clearer and have changed the formulation to the one suggested by the reviewer.*

- Line 184 to line 186: I believe you in what you have written, but the decoupling expression between CNGA and CNGB seems unclear to me in the Figure 5-B, where CNGA expression seems very low overall compared to the CNGB expression. This could be clarified by modifying the X-axis of the CNGA expression plot. I don’t see any problem of having different X-axis values for CNGA and CNGB.

*The point we want to make with the expression levels is that some species have much higher expression of CNGB than CNGA*, *which is unexpected from the current knowledge about subunit composition of functional channels. We believe this information would be lost if we changed the x axis range between different subunit genes in the same species.*

- Line 205: Why is the green spotted pufferfish not on the figure 8?
I would also suggest to remove this information from the main text and to place it in the figure 8 legend as it is not discussed and not useful for the study conclusions. Same goes for the Japanese pufferfish information on Line 253 to 256.

*This species is not included in figure 8 because the timetree.org database does not have data for this species. For this figure, only divergence times for species available in this database as stated in materials and methods, we have added a clarification about this in the materials and methods section.*

- Line 218 to 222: Redundancy of results with lines 239 to 256.

*This has been fixed.*

- Line 226: Change “To investigate the CNG gene repertoire further in a group of vertebrates where vision is a very important sense” to “To further investigate the evolution of CNG genes repertoire in a group of vertebrates with a rich and dynamic opsin gene repertoire (ref1, ref2, ref3) …”

Ref 1 : [https://www.ncbi.nlm.nih.gov/pmc/articles/PMC4617963/](https://www.ncbi.nlm.nih.gov/pmc/articles/PMC4617963/)
Ref 2 : [https://www.science.org/doi/10.1126/science.aav4632](https://www.science.org/doi/10.1126/science.aav4632)
Ref 3 : [https://doi.org/10.1146/annurev-cellbio-120219-024915](https://doi.org/10.1146/annurev-cellbio-120219-024915)


I disagree with the postulate that vision is a very important sense in actinopterygian. First, I believe that the rich opsin gene repertoire of these species is not due to a better vision than other vertebrates, but is due to the highly different light environments they face. Also, many fish species don’t rely at all on vision (cavefishes for example) and many species seems to rely much more on olfaction and taste for feeding, orientation or conspecific recognition. Thus, I also suggest to change this statement made on Line 118

*We have made changes to the text and cited ref 3 since it summarizes visual opsins well. We made the argument that vision is important in many lineages since they have retained many tandem duplicates of visual opsins. If vision wasn’t an important sense, these extra gene duplicates would be redundant. Actinopterygians, or more specific teleosts, is the only vertebrate lineage with this kind of expansions of the visual opsin genes.*

- Line 230: Replace “sturgeons that have seven genes” by “sturgeons and paddlefishes that have seven genes”

*This has been fixed.*

- Line 233: I would remove all those details about local duplications and simply write “in general, teleost fish have seven CNGA genes, with exceptions e.g. cyprinids and salmonids have experienced extra lineage specific WGDs, and some clade specific local duplications”

*This information is of importance to understand why the genes are the result of a tandem duplication and thus we would like to keep it.*

- Line 332: “rhe” -> “the”

*This has been done.*

- Line 381: If I understand correctly, you believe that the duplication of CNGB3 in teleost did not arise from the WGD, but by a local duplication. This seems unclear to me. What arguments do you have to differentiate between such a local duplication and the effect of WGD followed by a rearrangement?

*A tandem duplication rather than a rearrangement post 3R is supported by the same organization of the genes, namely proximity (usually less than 50 kbp) on the same chromosome and in the same orientation, in distantly related teleost species such as zebrafish and Asian arowana. This information is available in the results section and in table S1.*

- Line 389-390: The fact that all those species only have one CNGA4 excepted for Carassius auratus is interesting. Could you look at if in this species, one of the CNGA4 copy is a pseudogene but have not yet been erased from the genome? Also, I believe that it would be worth to mention somewhere in the manuscript or in the methods, that you don’t consider differences between complete genes and pseudogenes in your gene copy numbers. This is clearly visible on the datasupp2, where some genes are annotated as “LOW QUALITY PROTEIN” which are usually genes with frameshifts or premature stop codons in NCBI. This does not change your results and discussion as you focus on the overall gene repertoire and its evolution at a high evolutionary timescale (And it would need a lot of additional work to make a proper annotation of pseudogenes).

*The two CNGA4 sequences of goldfish show no evidence of being pseudogenes, both have RNA-seq reads and have no early stop codons. Thank you for this information, this information has been added to the materials and methods section.*

I also suggest to restructure the manuscript with this chapter order: 2.1 ; 2.3 ; 2.4 ; 2.5 ; 2.6 ; 2.7 ; 2.2. This would allow to first explain the evolution of CNGA and CNGB genes at the genomic level, and to then investigate their expression. With the actual chapter order, the transcriptomic analysis kind of break the flow by being placed in between the genomic investigations.

*We agree that this makes the flow better and has changed the text according to this suggestion.*

- Line 479: Please add details on the methods used for your manual annotation of genes.

*Sequences that were annotated manually or complemented was done so by using sequence homology and splice donor/acceptor sites. This information has been added to the materials and methods section.*

Methods sections 5.6 and 5.7: Did you make the synteny figures with a software? If so, indicate which software was used.

*No, the synteny images was made manually using Microsoft PowerPoint.*

Congratulations to the authors for this study, hoping that my suggestions, and those of the other reviewers, will help for the publication of this manuscript.

*Thank you, the suggestions by you and the other reviewer have been helpful and we think this makes the manuscript clearer.*
